# Research on Network Security Protection Technology Based on P2AEDR in New Low-Voltage Control Scenarios for Power IoT and Other Blockchain-Based IoT Architectures

**DOI:** 10.3390/s24216808

**Published:** 2024-10-23

**Authors:** Weiwei Miao, Xinjian Zhao, Nianzhe Li, Song Zhang, Qianmu Li, Xiaochao Li

**Affiliations:** 1 State Grid Jiangsu Electric Power Co., Ltd., Information & Telecommunication Branch; Nanjing 210024, China; miao_weiwei_sgcc@163.com (W.M.); zhaoxinjian_nj@163.com (X.Z.); m18362973161_1@163.com (S.Z.); 2 School of Computer Science and Engineering, Nanjing University of Science and Technology, Nanjing 210094, China; 3 School of Cyber Science and Engineering, Nanjing University of Science and Technology, Nanjing 210094, China; qianmu@njust.edu.cn (Q.L.); lixiaochao@njust.edu.cn (X.L.)

**Keywords:** low-voltage control, protection model, dynamic defense, trust assessment, deep learning, Internet of Things (IoT), Power IoT (PIoT), blockchain, Blockchain-based IoT (BIoT)

## Abstract

In the construction of new power systems, the traditional network security protection mainly based on boundary protection belongs to static defense and still relies mainly on manual processing in vulnerability repair, threat response, etc. It is difficult to adapt to the security protection needs in large-scale distributed new energy, third-party aggregation platforms, and flexible interaction scenarios with power grid enterprise systems. It is necessary to conduct research on dynamic security protection models for IoT and other Blockchain-based IoT architectures. This article proposes a network security comprehensive protection model P2AEDR based on different interaction modes of cloud–edge interaction and cloud–cloud interaction. Through continuous trust evaluation, dynamic access control, and other technologies, it strengthens the internal defense capabilities of power grid business, shifting from static protection as the core mode to a real-time intelligent perception and automated response mode, and ultimately achieving the goal of dynamic defense, meeting the security protection needs of large-scale controlled terminal access and third-party aggregation platforms. Meanwhile, this article proposes a dynamic trust evaluation algorithm based on deep learning, which protects the secure access and use of various resources in a more refined learning approach based on the interaction information monitored in the system. Through experimental verification of the dynamic trust evaluation algorithm, it is shown that the proposed model has good trust evaluation performance. Therefore, this research is beneficial for trustworthy Power IoT and other Blockchain-based IoT architectures.

## 1. Introduction

The new low-voltage control-related business is an important part of the new power system, which is mainly deployed in the company’s management information area and the Internet area. On the one hand, it is interconnected with the open network, and on the other hand, it is in direct contact with the controlled objects. Compared with traditional businesses, it has many access points, a small unit scale, a low voltage level, and uncontrollable physical security. The above characteristics of the new low-voltage control business bring new challenges to the traditional security protection system.

One is the widespread integration of large-scale distributed resources and third-party aggregation platforms into the power grid, with more complex access methods and explosive growth in the number of accesses. The existing network security grid, like deep protection architecture, is difficult to meet the security protection needs of large-scale control terminal access and third-party aggregation platforms, and there is an urgent need to improve the new active defense framework for security protection. Secondly, a low-voltage collection and perception application based on the Internet of Things (IoT) system has been established within the power grid to support business systems such as the distribution cloud master station, which has played a significant role in power grid data collection and perception. However, existing network security protection measures are aimed at meeting the needs of new low-voltage control business groups, and there are still shortcomings in user identity authentication and emergency response on the master station side, and there is room for improvement in threat monitoring and protection on the edge side. Third, the third-party aggregation business represented by electric vehicles and smart homes is booming. The third-party aggregation platform is deployed in the Internet cloud to participate in the regulation of a large number of user sources and load devices. It is urgent to build a safe interaction system with the grid business system, achieve global control of the grid over the third-party adjustable resources, and ensure the safe and stable operation of the power system.

The traditional network security protection mainly based on boundary protection belongs to static defense, which is difficult to adapt to the scenarios of new low-voltage control business cloud–edge interaction and cloud–cloud interaction. Therefore, research on dynamic security protection models is needed [1,2,3]. The P2DR model is one of the earliest active defense models applied in the field of network security, which mainly consists of four parts: security strategy, protection technology, detection tools, and emergency response [4]. However, the P2DR model ignores inherent variables such as personnel mobility, personnel quality, and instability factors in strategy implementation, as well as considerations of business system security requirements and protection costs, making it unsuitable for the security protection needs of new low-voltage control business scenarios. The zero trust architecture can to some extent compensate for the shortcomings of P2DR. Its core idea is that by default, no users/devices/systems inside or outside the network are trusted, and multi-factor identity authentication [5], continuous security evaluation, and dynamic access authorization are required to ensure the reasonable, legal, and compliant use of various resources in a refined manner [6].

Therefore, based on the P2DR model and zero trust architecture, this paper proposes a new network security comprehensive protection model, P2AEDR, for new low-voltage control business scenarios. Compared with P2DR and the zero trust framework, P2AEDR strengthens the defense capability in power grid business through continuous trust evaluation and dynamic access control, transforming from static protection to real-time intelligent perception and active defense and from “static” to “dynamic”. It can protect the normal execution of system functions while protecting system information, taking into account the overall availability and security of the system. It establishes an active defense system under the business, realizes real-time monitoring of network security, intelligent orchestration of security protection strategies, and precise prevention and control of the business, making up for the shortcomings of existing security models in internal defense and dynamic defense, and protects cloud edge interaction and cloud to cloud interaction in low-voltage control business scenarios, thus meeting the demand for secure interaction between large-scale control terminal access and third-party aggregation platforms.

## 2. Related Study

Early network security typically adopted a security concept centered around boundary protection, dividing the network into trusted and untrusted zones through security devices to achieve coarse-grained access control between different trust domains. However, in recent years, with the complexity of network security threats and the diversification and intelligence of attack methods, as well as the migration of mobile offices and various applications to the cloud, traditional physical boundary-based security models have exposed their own shortcomings [7,8,9,10,11].

In this environment, traditional boundaries evolve towards logical boundaries until they gradually disappear, and traditional trust models are broken. Zero-trust networks begin to emerge and quickly become a research hotspot in the field of security. Haddon [12] analyzed the concept, strategy, and implementation methods of zero-trust networks. Feng Jingyu et al. [13] proposed a zero-trust edge model to address the threat of compromised terminals in the context of the Power IoT (PIoT). Sellitto et al. [14] proposed a zero trust architecture for smart grids based on digital twin technology. Chen et al. [15] proposed a method combining blockchain [16,17,18,19,20,21] and zero trust to protect the PIoT [22,23,24,25,26,27,28,29,30,31,32]. Liu et al. [33] applied overlay network technology and zero trust security to the construction of enterprise information infrastructure.

In terms of security models, they have shifted from passive protection to active defense, emphasizing defense and recovery throughout the entire lifecycle. The PDR model is the earliest proposed security model that embodies this idea. In the late 1990s, the ANS Alliance established a new P2DR model based on the PDR model [34].

Traditional IoT security models typically focus on static protection, such as identity authentication, data encryption, and network isolation, which often fall short in the face of dynamic threats. In terms of vulnerability repair and threat response, manual disposal is still the core, which cannot be applied to the security protection of distributed and flexible access scenarios in new business applications. In contrast, P2AEDR integrates six modules, including real-time policy intelligent orchestration, dynamic security assessment, and automatic security response, forming a dynamic and intelligent security protection system.

The unique contribution of P2AEDR lies in its emphasis on real-time monitoring and response. Through the real-time state perception module, the system can instantly identify and respond to security events, ensuring that effective measures are taken quickly in the event of an attack. The dynamic security assessment module continuously evaluates network security risks based on real-time data, allowing protective measures to be adjusted in a timely manner according to changes in threats. At the same time, internal defense capabilities are strengthened by integrating internal changes in the system. This real-time performance and adaptability greatly enhance the security of IoT systems, especially in the face of complex and ever-changing attack environments; P2AEDR can provide more comprehensive and flexible protection strategies.

In summary, P2AEDR not only supplements the shortcomings of existing IoT security models in dynamic response and intelligent decision-making but also enhances overall security protection capabilities through comprehensive monitoring and optimization mechanisms, providing new ideas and methods for security management in IoT environments.

## 3. P2AEDR Model

In order to solve the security protection problem of a new low-voltage control-related business, this paper proposes the P2AEDR active defense model.

### 3.1. Overall Description

**Figure 1:**
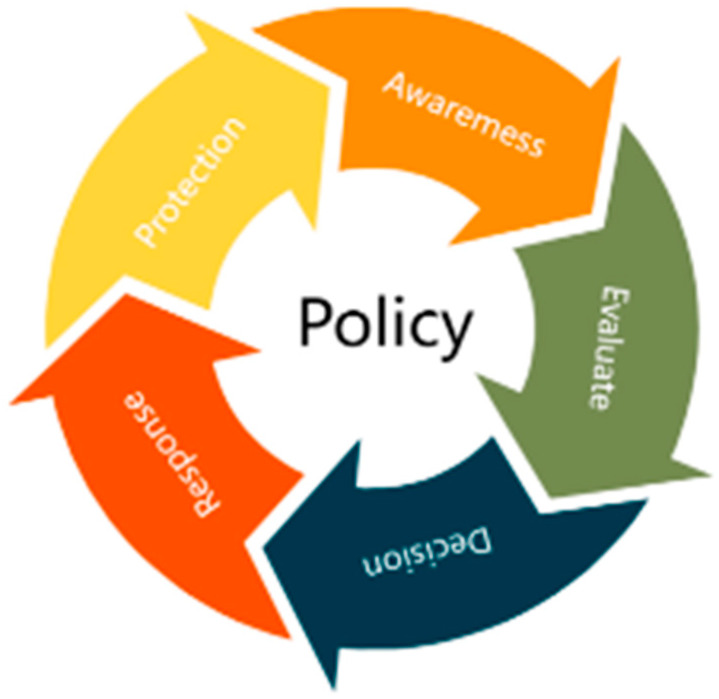
P2AEDR model diagram.

As shown in Figure 1, the P2AEDR model mainly includes six parts: real-time policy intelligent arrangement (Policy), static security protection (Protect), real-time state awareness (Awareness), dynamic security evaluation (Evaluate), intelligent security decision-making (Decision), and automatic security response (Response). The P2AEDR model adds a real-time state awareness module and a dynamic security evaluation module on the basis of the P2DR model, which can monitor the system operation status in real time and at the same time perceive the changes of internal factors of the business system in real-time to prevent hidden threats from occurring. The P2AEDR model adds a real-time state awareness module and a dynamic security assessment module on the basis of the P2DR model, which is able to detect changes in internal factors of the business system in real time while monitoring the system operation state in real time to prevent hidden threats from occurring, improve the automatic response capability of the system as a whole, realize the security control on low-voltage control resources, and enhance the internal defense capability of the system through multi-dimensional authentication of internal and external subjects, trust assessment, and dynamic authorization to enhance the internal defense capability of the system.

The six modules are closely linked and collaborate with each other, thus forming a set of secure, trustworthy, flexible, and dynamic defense systems in depth. Firstly, the real-time state perception module continuously monitors the network and system, detects abnormal behavior in a timely manner, and feeds back information to the real-time policy intelligent orchestration module to update and adjust security policies. At the same time, the static security protection module provides basic security measures to ensure the initial protection of IoT devices. The dynamic security assessment module conducts vulnerability analysis based on real-time monitoring data and transmits the assessment results to the intelligent security decision-making module, which combines internal and external intelligence to develop targeted response plans. The automatic security response module takes prompt action based on decision results, implements dynamic strategies and emergency measures, and ensures rapid response to security incidents. Ultimately, all modules form a closed loop through feedback mechanisms, continuously optimizing monitoring and response strategies to enhance overall network security situational awareness and proactive defense capabilities.

**Figure 2:**
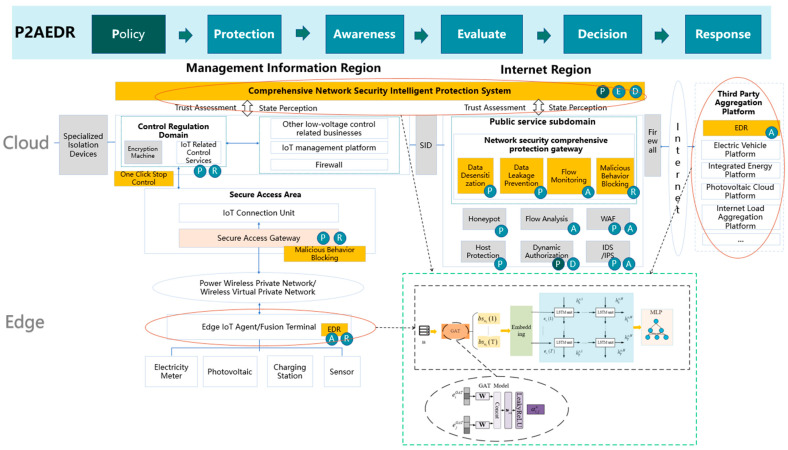
A novel security protection framework for low-voltage control-related operations based on P2AEDR. Among them, boxes represent solid objects or a certain link or module in the model structure, and different colors are used to distinguish importance, with darker colors indicating greater importance. The combination of circles and arrows points to the specific implementation principle of this module.

On this basis, this paper proposes a new low-voltage control-related business security protection framework based on P2AEDR, and the overall framework diagram is shown in Figure 2. On the basis of the existing network security protection system architecture, the network security boundary and partition are optimized to form a production control region, a management information region, and an Internet region. A security access platform is deployed in the management information region, and a security interaction platform is deployed in the Internet region, which are used to manage the access and interaction of internal and external network terminals and services and third-party aggregation platforms, respectively. Through real-time multi-dimensional identity authentication, subject–object environment security assessment, dynamic access authorization and control, dynamic security audit, and other means, it realizes dynamic security protection, dynamic trust management, dynamic approval and supervision of subject, object, behavior, and subject-related environment elements, and finally forms a safe, trustworthy, flexible, and dynamic deep defense system in the management information region and Internet region.

In Section 3.2 and Section 3.3, this paper details the use of the P2AEDR model in actual scenarios, including the grid management information area cloud edge interaction scenario and the Internet area cloud interaction scenario.

### 3.2. Management Information Region Cloud–Edge Interaction Scenario Application

**Figure 3:**
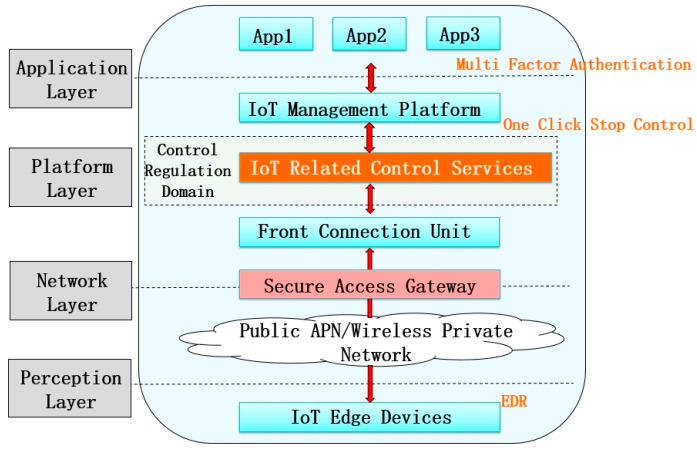
Management information region cloud edge interactive protection architecture diagram.

As shown in Figure 3, first, Endpoint Detection and Response (EDR) technology is used at the edge side by recording endpoint and network events, and this information is stored locally in an endpoint or centralized database. At the same time, a database of known Indicators of Compromise (IOCs) and behavioral analysis are combined to learn and monitor any possible security threats. After threat identification, an appropriate threat response is performed to isolate infected end devices, remove malware, block network connections, etc., to minimize the impact of the threat. Through the response, the infected end devices are repaired, security policies and rules are updated, and threat intelligence is shared to avoid being attacked by similar threats again.

Provincial (prefectural and municipal) power grid enterprises coordinate the setting of network access points in the management information area and uniformly construct security access gateways to access various types of IoT terminals. At the same time, new control-related regulatory domains are added in the management information region to physically isolate the IoT management platform and the security access area and logically isolate the control-related business from the non-control-related business; measures such as multi-factor authentication and login and one-key stopping and control are used to realize the security protection of the IoT management platform and the master station and to improve the capacity of emergency protection, in accordance with the idea of “control separation”. According to the idea of “separation of management and control”, the commands involving grid control in the IoT control-related business need to be sent to the terminal side for execution through the control-related regulation domain, and the regulation and control commands of the identification business are encrypted to ensure the security of the command transmission and execution process, so as to effectively prevent the destruction of the control-related business by malicious attacks and illegal operations and to realize the mechanism and process of the control-related commands and support the low voltage distributed. It realizes the mechanism and process of issuing control-related commands, supports the safe issuance and execution of single and group control commands for low-voltage distributed resources such as photovoltaic, smart switch, etc., ensures the safe operation of low-voltage microgrids, improves the security protection level, and supports the business of single-point control and group control.

**Figure 4:**
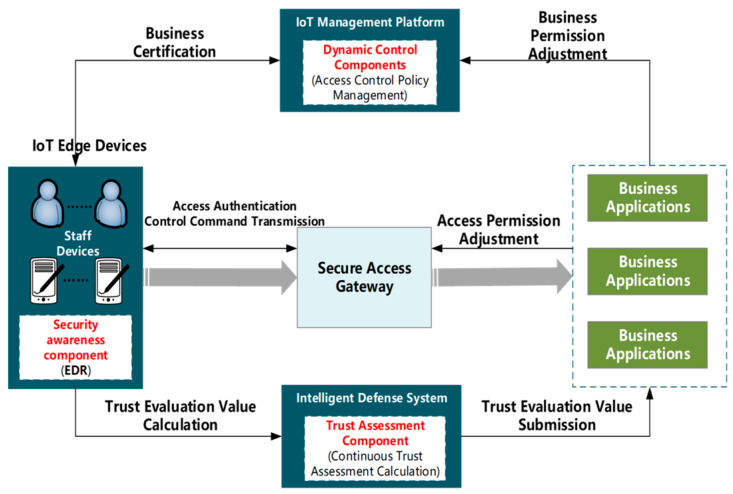
Management information region integrated cybersecurity intelligent defense system.

Meanwhile, a comprehensive network security intelligent defense system is designed in this paper, and the specific process is shown in Figure 4. Traditional access control is based on network entity definition and pre-assigned binary determination policy, which usually utilizes static authorization rules as well as black-and-white lists for one-time assessment of business access. In contrast, in the P2AEDR model, a deep learning-based trust assessment component is proposed, which is able to perform continuous trust value assessment by learning the behavior of the IoT edge devices based on the abnormal behavior data continuously reported by the EDR and monitored on the gateway side and reporting the trust value to the business application system. By combining the static access control component with the dynamic trust assessment component, the authorization range of the subject accessing the low-voltage control-involved business system will be constantly changing according to the influence of past and current behaviors, identity information, and the network environment, etc. The minimum authorization is dynamically implemented for each business access, which solves the problem of insufficient dynamism of the security policy under the traditional static control mechanism and strengthens the ability of the network to defend against threats.

### 3.3. Internet Region Cloud–Cloud Interaction Scenario Application

**Figure 5:**
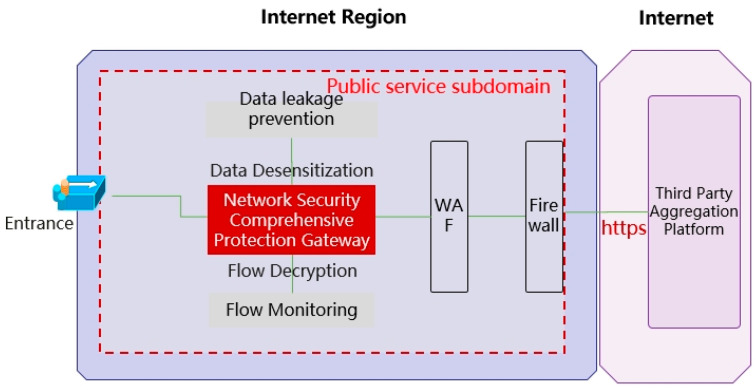
Internet region cloud interactive protection architecture diagram.

In the cloud interaction protection architecture of the Internet Region Cloud, analyze and classify the characteristics of the interaction between the third-party aggregation platform and the power grid business, based on the classification from the three aspects of the security interaction mechanism, network interaction behavior restoration and anomaly monitoring, and data interaction content security monitoring, carry out continuous risk monitoring of the interaction behavior anomalies, data flow anomalies, API interface access anomalies, etc., and dynamically control access privileges of the third-party aggregation platform based on the trust level. Based on the trust level, it dynamically controls the access privileges of the third-party aggregation platform and builds the border security interaction protection capability of the public service subdomain of the power grid and the third-party aggregation platform for safe interaction. The overall protection architecture is shown in Figure 5.

First of all, build a public service sub-domain for the safe interaction between the grid business and the third-party source platform, and set up a comprehensive protection gateway in the safe access area of the public service sub-domain, which is mainly responsible for traffic monitoring, data leakage detection, and malicious behavior blocking of the interaction behavior of the third-party aggregation platform, proactively discovering various types of security events in the process of data interaction, and carrying out in-depth digging and security correlation analysis of the data. By means of access flow and interface call, data security capabilities are reasonably orchestrated into the data flow process, thus realizing active protection, linkage protection, and accurate response to different security risks through security policies.

**Figure 6:**
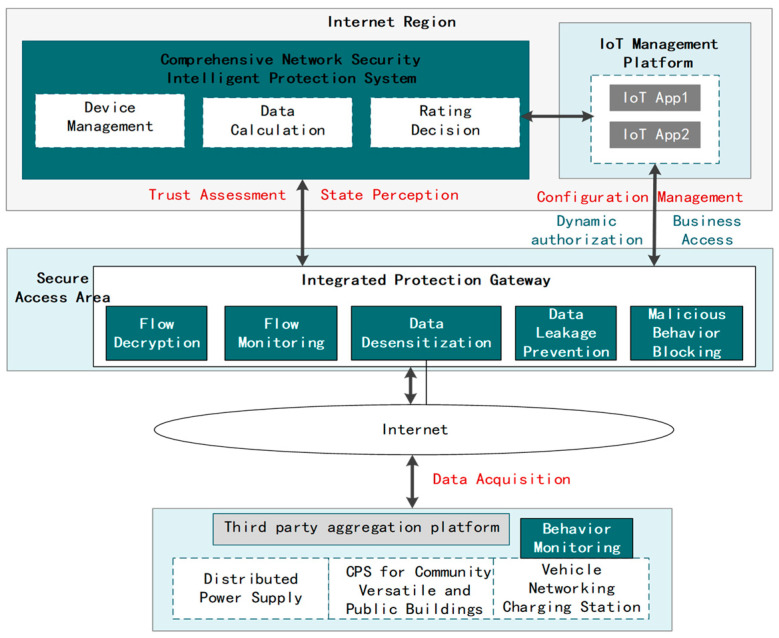
Dynamic authorization process for Internet regions.

The interaction between the third-party aggregation platform and the grid business system is affected by multi-dimensional factors such as communication protocols, business interaction requirements, etc. It is difficult to discover potential anomalies by simply considering the network layer attribute characteristics, such as protocol compliance of the interaction. Therefore, this paper constructs a fine-grained resource dynamic access authorization mechanism based on the security trust degree of interacting subjects on the basis of traditional access control, and the overall process is shown in Figure 6. The comprehensive network security intelligent defense system integrates the historical interaction behavior of the third-party aggregation platform, network environment factors, and identity information to generate the security trust degree, according to the third-party aggregation platform’s security trust degree, adopts the minimized security access control policy, combines the short-time token technology to realize the dynamic access authorization of a single session, solves the problem of insufficient dynamism of the security policy under the traditional static control mechanism, and enhance the ability of network defense against threats so as to build the security interactive protection capability of the power grid and the third-party aggregation platform.

## 4. Algorithm Design of the Core Module

In order to meet the needs of complex large-scale access entities for continuous trust evaluation and dynamic access control in low-voltage control-related scenarios, this paper adds a new trust evaluation algorithm based on deep learning to the comprehensive network security intelligent defense system. This algorithm is applied to the dynamic security assessment module in the P2AEDR model. Through real-time monitoring of behavior data by the real-time state perception module, it dynamically evaluates the security of interactive subjects and provides a basis for judgment for the automatic security response module. The overall process is shown in Figure 2, mainly consisting of three parts: the access subject learning module, the sensitive data protection module, and the interactive behavior learning module.

### 4.1. Edge Subject Learning Module

Through monitoring techniques such as edge-side EDR, the behavioral records of different access subjects can be obtained for learning, but the impact of different access subjects on the trust assessment model is different. For example, some edge devices are important confidential devices, and the interaction behaviors of these subjects should be more valuable for learning. Specifically, this paper proposes an access subject learning module to learn the weights between access subjects and quantify them as the degree of contribution of the current access subject to the overall model training.

In the access subject learning module, this paper draws on the weight learning approach in Graph Attention Network (GAT) [35]. The self-attention mechanism is incorporated in GAT. For the target node i, the importance of each of its neighbors can be determined using the self-attention mechanism. Specifically, for the trust relationship pair $\left(u_{i},u_{j}\right)$, their attention scores are as follows:

(1) $$z_{i,j}^{u}=Attention(W_{1}e_{i}^{GAT},W_{1}e_{j}^{GAT})$$

where $z_{i,j}^{u}$ is a scalar representing the attention weight, $W_{1}\in R^{d\times d}$ is a linear mapping matrix, and Attention is the attention layer in GAT. Here, this section treats the connection between access subjects as an undirected graph, where $u_{j}\in Nei(u_{i})$, i.e., $u_{j}$, is a first-order neighbor of the current node $u_{i}$.

Also, a weight vector $a^{T}$ is used to parameterize the feedforward neural network, and LeakyReLU is chosen as the activation function so that Equation (1) can be expressed as follows:

(2) $$z_{i,j}^{u}=LeakyReLU(a^{T}[W_{1}e_{i}^{GAT}||W_{1}e_{j}^{GAT}])$$

where $a^{T}$ denotes the transpose of the parameters of the attention layer in GAT, and || denotes the concatenation operation of the vectors.

After that, the Softmax activation function is used to calculate the importance values of all neighbors of the target node $u_{i}$ with the following formula:

(3) $$\alpha_{i,j}^{u}=Softmax\left(z_{i,j}^{u}\right)=\frac{\exp(z_{i,j}^{u})}{\sum_{u_{j}\in Nei(u_{i})}^{U}\exp(z_{i,j}^{u})}$$

where $\alpha_{i,j}^{u}$ is the final attentional weight value output by GAT, i.e., the importance value of $u_{j}$ to $u_{i}$, and exp denotes the exponential function.

### 4.2. Sensitive Data Protection Module

There exists a large amount of sensitive data in the low-voltage control-related business scenarios. In order to prevent the leakage of sensitive information of nodes during the trust assessment process, this section proposes a sensitive data protection module, which randomly adds virtual interaction information for each access subject on the basis of the original interaction behavior, connecting different local blocks, to prevent attackers from carrying out attribute inference attacks, and to protect the sensitive data of electric power.

Based on the access subject learning module, this section uses the learned subject weights for the construction of virtual interaction behavior. For the current node $u_{i}$, first randomly select some access information, $I_{i}^{without}\subseteq I/I_{i}$, which it does not have, and then construct the virtual interaction behaviors to be added to ${u_{i}}^{’}s$ behavioral information through the interaction information of all the first-order neighboring subjects $u_{j}$. The weight of the contribution of the accessing subject $u_{j}$ to the current behavioral information is $\alpha_{i,j}^{u}$, and the specific construction formula is as follows:

(4) $$r_{i,v}^{virtual}=\sum_{u_{j}\in Nei(u_{i})}^{U}\alpha_{i,j}^{u}r_{j,v}$$

where $r_{i,v}^{virtual}$ is the virtual behavioral information added by $u_{i}$, and $r_{j,v}$ is the real-existing behavioral information of a ${u_{i}}^{’}s$ first-order neighboring subject $u_{j}$, with $v\in I_{i}^{without}$. It is necessary to specify that the most recent temporal information in the neighboring subject is chosen as the time of the newly added behavioral information.

### 4.3. Interactive Behavior Learning Module

In order to be able to perform continuous trust assessment and dynamic access control for different low-voltage control-related business access subjects, this section performs feature learning of low-voltage control-related access subjects on the basis of the interaction behavior relationship graph constructed by the above two modules. In fact, the interaction records between subjects can be regarded as a sequence with time information, and in this paper, these time sequences are divided into multiple slices, and the long and short-term dependency information between the slices is dynamically captured through the Long Short-Term Memory (LSTM) network model, which makes the trust assessment traceable and thus assesses the trust relationship between nodes more accurately.

First, the state update value ${\tilde{c}}_{t}$ corresponding to the current time slice t, the input gate $i_{t}$, the forgetting gate $f_{t}$, and the output gate $o_{t}$ are computed by inputting the access body embedding matrix into the LSTM cell as follows:

(5) $$\left[\begin{array}{c} {\tilde{c}}_{t}\\ i_{t}\\ f_{t}\\ o_{t} \end{array}\right]=\left[\begin{array}{c} tanh\\ \sigma \\ \sigma \\ \sigma \end{array}\right](W_{2}\left[\begin{array}{c} e_{u_{i}}\\ h_{t-1} \end{array}\right]+b)$$

where σ is the Sigmod activation function; $e_{u_{i}}$ is the embedding feature matrix of node $u_{i}$ sliced at time t; $W_{2}$ is the weight matrix; and b is the bias matrix.

After obtaining ${\tilde{c}}_{t}$, $i_{t},\;f_{t}$, and $o_{t}$, the current cell state $c_{t}$ can be calculated as follows:

(6) $$c_{t}=f_{t}☉c_{t-1}+i_{t}☉{\tilde{c}}_{t}$$

where ☉ is a dot-multiplication operation.

Finally, the final embedding $h_{t}$ of the current time slice t can be obtained from the current cell state $c_{t}$ and the output gate $o_{t}$ as follows:

(7) $$h_{t}=o_{t}☉\tanh(c_{t})$$

One advantage of using the LSTM network is that it is free to increase the horizontal width and vertical depth of the network. H denotes the vertical depth of the LSTM network and T denotes the number of time slices and the horizontal width of the LSTM network. Finally, for nodes $u_{i}$ and $u_{j}$, the learned feature embedding can be expressed as follows:

(8) $$\left\{\begin{array}{c} H_{u_{i}}=\frac{h_{1}^{iH}+h_{2}^{iH}+\ldots +h_{T}^{iH}}{T}\\ H_{u_{j}}=\frac{h_{1}^{jH}+h_{2}^{jH}+\ldots +h_{T}^{jH}}{T} \end{array}\right.$$

The LSTM network can fuse multiple sparse features to obtain the time series characteristics of the service access subjects, capture the key information of the corresponding time slice through the memory unit, and incorporate these key pieces of information into the node state at the current moment. In this process, the access subjects can remember behavioral events that have a greater impact on them, thus forming a more accurate trust relationship. At the same time, new interaction behavior records of access subjects can be added in real time for continuous dynamic trust evaluation.

### 4.4. Trust Assessment Module

Through the interaction behavior learning module, the feature embedding of the low-voltage control-involved access subject can be dynamically obtained. After obtaining feature embedding, this section will carry out the evaluation of the trust relationship and take the trust evaluation value as an important reference factor for subsequent dynamic access control. Specifically, the trust assessment calculation formula is as follows:

(9) $$\tilde{r}=H_{u_{i}}☉H_{u_{j}}$$

where ☉ is a dot product operation and $\tilde{r}$ is the trust assessment value.

After obtaining the trust assessment value $\tilde{r}$ of $u_{i}$ and $u_{j}$ through the calculation of Equation (9), this section sets a trust relationship threshold; when the trust assessment value is greater than or equal to threshold, it is considered that there is a trust relationship between the two nodes $u_{i}$ and $u_{j}$. When the trust assessment value is less than threshold, it is considered that there is a distrust relationship between the two nodes $u_{i}$ and $u_{j}$. In order to optimize the model proposed in this paper, the binary cross entropy function is used as the loss function in this section, which is calculated as follows:

(10) $$Loss=-\sum_{\left(u_{i},u_{j}\right)}[Trust\left(u_{i},u_{j}\right)\log\left(\hat{T}rust\left(u_{i},u_{j}\right)\right)\;\;\;\;\;\;\;\;\;\;\;\;+\left(1-Trust\left(u_{i},u_{j}\right)\right)\log(1-\hat{T}rust\left(u_{i},u_{j}\right))]$$

where $\left(u_{i},u_{j}\right)$ denotes two nodes in the dataset that have a trust or distrust relationship. $\hat{T}rust\left(u_{i},u_{j}\right)$ is the trust value evaluated by the model and $Trust\left(u_{i},u_{j}\right)$ is the true trust and distrust value in the dataset; if the two nodes are in a trust relationship, then $\hat{T}rust\left(u_{i},u_{j}\right)$ is taken to be 1, or 0 if the two nodes are in a distrust relationship.

## 5. Experiment and Result Analysis

In this paper, the Epinions dataset [36], which has similar characteristics to the grid interaction behavior data, is selected as the experimental dataset, and a large number of experiments are designed on the Epinions dataset to evaluate the effect of the dynamic trust assessment module in the proposed P2AEDR framework.

### 5.1. Experimental Settings

(1) Dataset selection: as shown in Table 1, the Epinions dataset records the subject’s own information, the trust relationship information between subjects, and behavioral information (interaction information, rating information, time information, etc.), and the rating in the dataset ranges from 1 to 5. In the experiment, this paper does not consider the size of the specific rating value and instead regards the record with a rating as a record of an interaction. Meanwhile, nodes with more than 10 cores are screened for training in this paper.

(2) Parameter setting: the parameters designed in the experiment are virtual access information adding ratio *p*, trust evaluation threshold, learning rate parameter learning_rate, training set size training_size, dropout layer ratio dropout.

(3) Evaluation metrics: According to the literature [37], the experiments use F1-score and Accuracy as evaluation metrics, where the larger the value of the experimental results, the better the performance of the model.

(4) Comparison methods: In order to demonstrate and confirm the validity and feasibility of the models proposed in this paper, this section selects some representative methods and models for comparison, which are described as follows:

- CPSRP [38]: CPSRP mainly applies the r-GRU network and MLP network to extract the time series features of subjects and predict the trust relationship between subjects. Meanwhile, the model uses the improved Simhash technique and differential privacy technique to protect privacy and security.
- SHLP [37]: SHLP only uses scoring information from a single platform to predict the trust relationship and type of relationship between subjects and does not consider the integration of subject information from multiple platforms.
- JC [39]: Common features are extracted from historical behavioral records using the subject’s rating vectors, but the method does not take into account the temporal information in the historical behavioral records.
- This paper’s method (DLTEM): This paper’s method simultaneously combines the access subject learning module, the interaction information construction module, and the historical behavior learning module for trust relationship assessment.
- Variant method (DLTEM-VR): On the basis of the method in this paper, the access subject learning module and the interaction information construction module are removed, and the trust relationship assessment is carried out only by learning behavioral information.

### 5.2. Experimental Results

In order to verify the effectiveness and feasibility of the method proposed in this article, experimental tests were conducted to evaluate the accuracy of the above comparative methods on the Epinions dataset. The experimental results are shown in Table 2, where three sizes of {40%, 60%, 80%} were selected for the training set size ratio, and the accuracy of the method trust prediction was obtained. The bold part represents the optimal result in the current situation.

The experimental results show that the accuracy of the method in this paper is optimal for prediction under each training set size, and the accuracy improvement is even greater in the case of small training sets. JC focuses only on a single attribute of the data and ignores the time-series features of the subject, which leads to poor performance. Although CPSRP utilizes the r-GRU network to capture the time-series features of the subject, the perturbation techniques used in CPSRP (including improved Simhash and differential privacy) severely damage the time-series features of the subject’s original data, which results in lesser performance than the method proposed in this paper. SHLP uses only interaction information to learn features and does not take into account the information of the trust network structure while also using Simhash for privacy preservation, which leads to performance degradation.

The results of the ablation experiments show that the overall performance of the ontology method is still significantly better than the variant method. The ontology method protects the subject data’s privacy and security and alleviates the uneven distribution of data based on the variant method. At the same time, the present method does not have an impact on the accuracy of trust assessment but rather has some slight improvement. Therefore, it can be concluded that the access subject learning module and the interaction information construction module in the method proposed in this paper can maintain good prediction accuracy while protecting the privacy and security of the access subject and mitigating the data sparsity. At the same time, we use formal methods to verify the correctness of blockchain code, effectively reducing the occurrence of errors and security vulnerabilities [40,41].

### 5.3. The Effect of the Hyperparameter p on the Experiment

In this section, the effect of the virtual interaction record allocation ratio *p* on the trust assessment model is discussed. The model proposed in this paper first calculates the weight between different subjects by accessing the subject learning module and then uses this weight to add virtual interaction information in the interaction information building module. Added interaction information can be used to prevent the attacker from inferring the attack and, at the same time, alleviate the problem of uneven data distribution to a certain extent. The proportion of added virtual information is determined by the parameter *p*. The larger the value of *p*, the more virtual information will be added for each subject, and the smaller the value of *p*, the less virtual information will be added for each node. In the experiments, this section sets the value of *p* to {0.1, 0.3, 0.5, 0.7}, sets the training set size to {40%, 60%, 80%}, and records the experimental results of F1-score and accuracy in each case.

**Figure 7:**
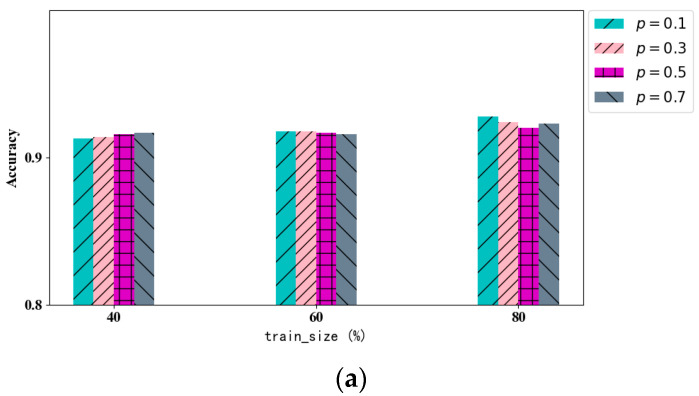

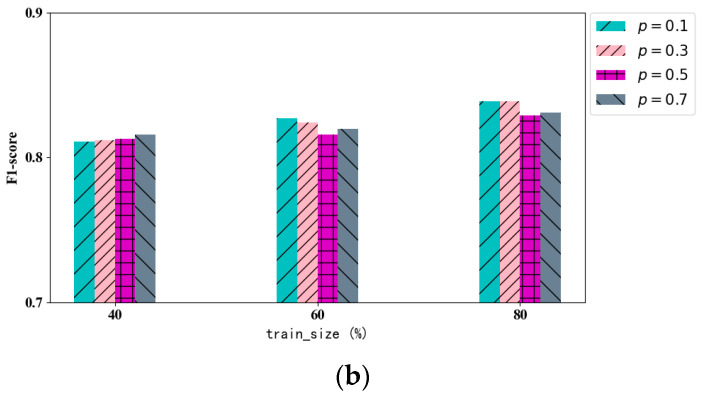
Add plots of the results of scale-*p* experiments, where (**a**) denotes the experimental results of accuracy and (**b**) denotes the experimental results of F1-score.

The specific experimental results are shown in Figure 7, where the optimal situation of each metric in each column of the table is indicated in bold. In Figure 7a, the graph records the change in accuracy with the increase in training set size under different *p* values, and in Figure 7b, the graph records the change in F1-score with the increase in training set size under different *p* values. From the experimental results, it can be found that both accuracy and F1-score indicators can be maintained in a certain interval under different *p* values, which indicates that the virtual interaction information adding module proposed in this paper can not only enhance the security of the data but also maintain the evaluation accuracy of the original model. Meanwhile, under different training set sizes, different *p* ratios have different effects on the experimental results. In the case of 40% training set size, because the training set size itself is relatively small and the data are relatively sparse, a higher value of *p* will improve the accuracy of training more. In the case where the training set size is between 60% and 80%, because the training set size is relatively large and the data are relatively numerous, a lower value of *p* is more favorable for model training and accuracy improvement. Therefore, it is more favorable for the model’s accuracy to select the appropriate proportion of virtual information added according to the size of the actual dataset.

### 5.4. The Effect of the Hyperparameter Threshold on the Experiment

In this section, the impact of the trust relationship judgment threshold on the trust assessment model is further investigated. Theoretically, setting a higher threshold in the model will lead to a stricter formation of trust relationships between access subjects, and a smaller threshold will lead to an easier establishment of trust relationships between access subjects. The experiments set the threshold as {0.3, 0.4, 0.5, 0.6, 0.7}, set the training set size as {40%, 60%, 80%}, and recorded the results of the F1-score and accuracy experiments in each case, respectively.

**Figure 8:**
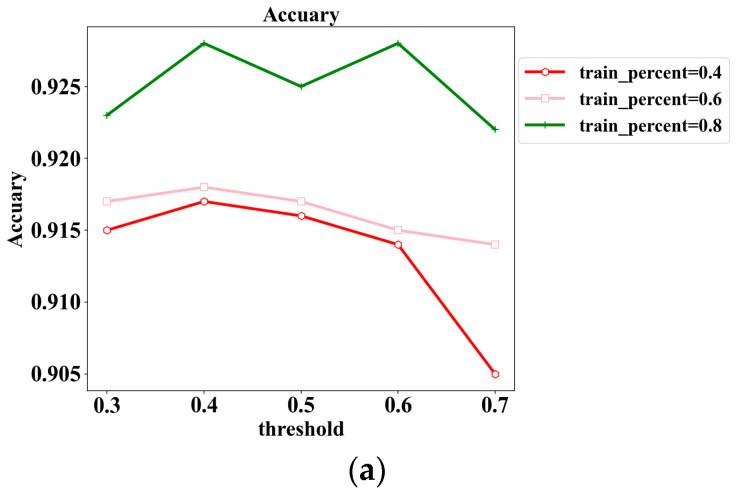

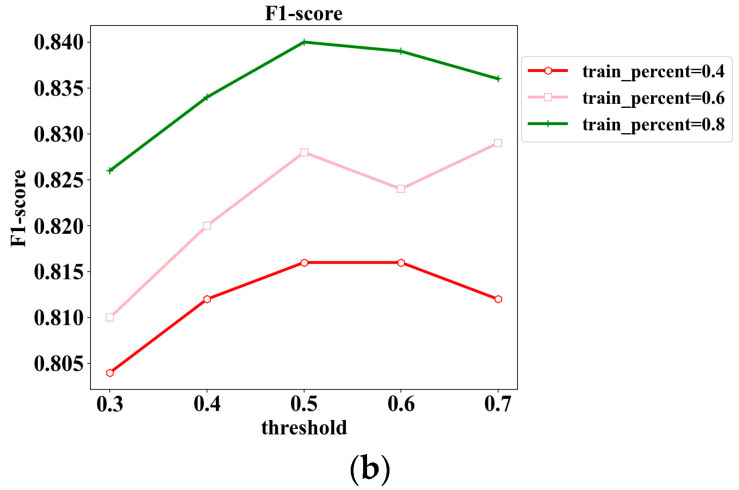
Threshold experimental result graph, where (**a**) denotes the experimental result of accuracy and (**b**) denotes the experimental result of F1-score.

The specific experimental results are shown in Figure 8, where the optimal case of each metric in each column of the table is indicated in bold. In Figure 8a, the graph records the change in accuracy with increasing threshold under different training set sizes, and in Figure 8b, the graph records the change in F1-score with increasing threshold under different training set sizes. From the experimental results, it can be found that both accuracy and F1-score are basically the best case when the threshold is taken as 0.5 and the model reaches the best performance. At the same time, the model performance is also better when the threshold is taken at 0.4 and 0.6 compared to the cases when the threshold is taken at 0.3 and 0.7. Therefore, appropriately setting a balanced threshold can significantly improve the accuracy of trust assessment of access subjects, and it is not the case that a higher or lower threshold is better for the experimental results.

## 6. Discussion on the Practical Application of the P2AEDR Model

### 6.1. Application and Promotion Channels

The specific implementation of this framework follows the principle of “overall planning, gradual promotion, and pilot first”, and applies and promotes research results. The first stage is a small-scale pilot project. Conduct pilot applications in the participating units of this project, using a typical new type of low-voltage control business as the application scenario, verify the project results, and conduct comprehensive evaluations to form pilot experience. The second stage is scale application. On the basis of previous pilot experience, expand the pilot scope, apply it on a large scale, improve and enhance technology, improve system performance, reduce system costs, enhance platform management performance, gain large-scale application experience, and form relevant standards. The third stage is comprehensive promotion. Based on the experience of scale application, carry out standardization construction, expand pilot projects, and gradually promote their application in the power industry. At the same time, a feedback system will be established to broadly collect experiences, issue reports, and improvement suggestions from pilot enterprises and users during the expansion of the pilot scope, and optimizations will be made based on this feedback.

### 6.2. Analysis of Costs and Benefits

The costs associated with the implementation of P2AEDR encompass multiple aspects, including technology investments (such as hardware, software, and cloud service fees), human resources (recruiting cybersecurity experts and employee training), operational maintenance (ongoing expenses for daily system maintenance and incident response), compliance and certification (costs for compliance audits and security certifications), risk management (expenses for vulnerability remediation and cyber insurance), as well as project management and testing evaluations during the implementation phase. While the initial investment may be significant, the long-term benefits of enhanced cybersecurity, reduced potential losses, and optimized business processes can significantly mitigate the economic risks associated with security incidents and improve overall operational efficiency.

Regarding benefits, this paper studies the key technologies for network security protection of new low-voltage control services, strengthens the security of IoT control services accessed through cloud edge interaction and third-party aggregation platform services accessed through cloud to cloud interaction, and achieves the goal of enhancing the autonomous protection and active defense capabilities of the power grid system. The relevant technological achievements can not only improve the security protection system of the new power system and guide various control edge devices and third-party aggregation platforms to securely access the company’s network, but also extend the security protection capabilities to the security monitoring of various heterogeneous power business terminals and systems, forming a powerful supplement to the company’s existing network security defense system.

The P2AEDR model can be validated through pilot testing and applied in practice, bringing considerable economic benefits. It can effectively reduce the response time of new low-voltage control business network security incidents, improve the efficiency of network security incident handling, reduce the cost of security management manpower, and help the smooth construction and safe operation of business systems, which can bring indirect economic benefits. After the promotion and implementation of the P2AEDR model, it can effectively prevent network attacks initiated by edge devices and third-party aggregation platforms, improve the analysis, early warning, self-healing, and disaster prevention capabilities of the power grid system, effectively reduce the economic losses caused by network attacks, and bring potential economic benefits.

Meanwhile, the P2AEDR model can bring significant social benefits. The implementation of P2AEDR will provide strong support for the construction and improvement of the security protection capabilities of the new power system and provide basic support for the trusted control, threat analysis, and linkage disposal of low-voltage control business access. It will timely detect anomalies in control edge devices and third-party aggregation platforms and issue warnings, which has profound significance for the construction and improvement of the proactive defense system of the State Grid Corporation of China. The P2AEDR model complies with the relevant policies of the country to strengthen the construction of the basic information network security guarantee system, meets the network security construction needs of the State Grid Corporation of China, and plays a demonstrative role in the network security protection work of the national energy and other industries.

### 6.3. Analysis of Scalability

The scalability of the P2AEDR model is reflected in six key aspects: modular design, dynamic policy adaptation, efficient data processing capabilities, integrable interfaces, continuous learning and optimization, and support from the community and ecosystem. Its six modules are built on a modular architecture that allows for easy addition of new features or expansion of existing modules as needed, enabling the system to flexibly respond to the integration of new devices and technologies. The real-time intelligent orchestration and dynamic security assessment modules can dynamically adjust security policies based on changes in the network environment and the addition of devices, ensuring the system can quickly address new security threats while optimizing resource allocation.

As the number of devices increases, data traffic will also rise significantly. Therefore, P2AEDR possesses efficient data processing capabilities to support high-frequency data analysis in the real-time status perception and dynamic security assessment modules. This can be achieved by introducing distributed computing, edge computing, or cloud computing technologies to enhance the system’s processing power. Additionally, P2AEDR should provide standardized APIs and interfaces to facilitate easy integration with various types of devices and systems, promoting interoperability with other IoT platforms and supporting connections among diverse devices and services, thereby enhancing the system’s scalability. Through the intelligent security decision module, P2AEDR utilizes machine learning and artificial intelligence technologies to continuously optimize security strategies based on historical data and user feedback, ensuring efficient security protection amid an increasing number of devices and complex interactions. Moreover, by establishing a community for users and developers, P2AEDR can gather user needs and feedback, quickly adapt to market changes, and promote the continuous development and expansion of the model, ensuring its long-term effectiveness.

Additionally, the P2AEDR model can effectively integrate with current security systems through multiple steps to enhance overall security. First, a comprehensive evaluation of the existing security system is necessary to identify security measures and potential vulnerabilities, determining the areas where the P2AEDR model can supplement existing solutions. Next, modular integration can be gradually implemented, starting with key modules to ensure coordination with the existing system. By establishing a unified security policy template, the strategies of P2AEDR can be aligned with the security measures of the existing system. Furthermore, utilizing programmable interfaces enables integration with existing devices (such as firewalls and intrusion detection systems), facilitating real-time information sharing and automated responses. Data sharing and integration enhance monitoring capability for potential threats, while the intelligent security decision module conducts in-depth analysis of existing data to formulate effective response strategies. Additionally, a continuous monitoring mechanism can be established, leveraging P2AEDR’s automated security response capabilities to promptly address security incidents and continuously optimizing security strategies based on feedback. Finally, training relevant personnel will enhance their understanding of the P2AEDR model and overall security awareness, thereby forming a more comprehensive, dynamic, and efficient security protection system capable of effectively responding to the ever-evolving cybersecurity threats.

In summary, the P2AEDR model possesses robust scalability, enabling it to maintain efficient and flexible security protection in the face of an increasing number of devices and interactions through modular design, dynamic adaptation, efficient data processing, standardized interfaces, continuous learning, and ecosystem support.

### 6.4. Limitations and Challenges

Our experiment using the Epinions dataset raises questions about generalization to IoT. However, when using non-IoT datasets, the P2AEDR model may face several limitations. Firstly, these datasets often lack features specific to particular IoT devices, resulting in suboptimal performance when identifying security threats. Secondly, non-IoT datasets are typically static, failing to reflect the dynamic changes in IoT environments, which limits the model’s real-time threat detection capabilities. Additionally, these datasets may not include unique data characteristics inherent to IoT, complicating feature engineering. Furthermore, the limited attack scenarios covered in non-IoT datasets do not comprehensively address the specific threats posed by IoT, ultimately impacting the model’s effectiveness.

Applying the P2AEDR model in heterogeneous IoT systems also presents a range of challenges. The diversity of devices complicates data integration and analysis, increasing the difficulty of achieving unified security policies. The significant volume of real-time data generated poses a challenge to the model’s processing capabilities, necessitating efficient analysis of high-frequency data streams. Moreover, the lack of standardized protocols may lead to compatibility issues with various devices and systems. Additionally, the dynamic nature of devices and networks in heterogeneous environments requires P2AEDR to possess the flexibility to adjust security strategies, which often depends on real-time data analysis and intelligent decision-making, thereby increasing the model’s complexity and computational resource demands. In future work, we will further improve the flexibility of our models and better apply them to different scenarios and systems.

## 7. Conclusions

In the new low-voltage control-related business scenarios of the power grid, this paper proposes a new network security protection model, P2AEDR, which mainly consists of six parts: real-time policy intelligent orchestration, static security protection, real-time state awareness, dynamic security assessment, intelligent security decision-making, and automatic security response. This paper introduces the specific application of the P2AEDR framework from the two business divisions of management information region and interconnection region in the low-voltage control business, which shifts the original network security protection from the model centered on static protection to the model with real-time intelligent sensing and automated response and changes the “static” into “dynamic” to protect the low-voltage control business. It shifts the original network security protection from a static protection core model to a real-time intelligent sensing and automated response model, changes “static” into “dynamic”, protects cloud–edge and cloud–cloud interactions in low-voltage control-related business scenarios, and meets the needs of large-scale control-related edge terminal access and third-party aggregate platform access security protection.

At the same time, this paper proposes a new type of trust assessment algorithm based on deep learning and edge computing, which can be based on real-time monitoring of the subject interaction behavior through the access subject learning module, sensitive data protection module, and interactive behavior learning module to learn the characteristics of different access subjects in the business so as to calculate the trust value of the access subject and to protect the safe access and use of resources in a more refined learning way and, at the same time, accelerate the training process through edge computing. Finally, this paper verifies the effectiveness of the proposed dynamic trust assessment algorithm through experiments, and the assessment accuracy in different experimental situations is higher than that of the baseline model, with good stability and feasibility. Hence, this research is beneficial for trustworthy Power IoT and other Blockchain-based IoT architectures.

In our future work, we aim to conduct further testing across a variety of real-world scenarios to comprehensively evaluate the costs and benefits associated with our model. This will involve assessing performance metrics in diverse applications to ensure robustness and applicability. Additionally, we plan to refine different modules of our system based on the insights gained from these evaluations. By iteratively improving our approach, we seek to enhance both the efficiency and effectiveness of our solution, ultimately leading to a more versatile and impactful model.

## Figures and Tables

**Table 1:** Statistical information on the Epinions dataset.

Causality	Numerical Size
Number of access subjects	85,000
Number of interaction records	13,668,319
Number of relationships of trust	841,372

**Table 2:** Experimental results.

Method	Training Set Size		
	40%	60%	80%
CPSRP	0.894	0.903	0.911
JC	0.545	0.560	0.612
SHLP	0.885	0.906	0.918
DLTEM-VR	0.910	0.916	0.923
DLTEM	**0.917**	**0.918**	**0.928**

## Data Availability

Data are contained within the article.
